# Cognition and the Default Mode Network in Children with Sickle Cell Disease: A Resting State Functional MRI Study

**DOI:** 10.1371/journal.pone.0157090

**Published:** 2016-06-09

**Authors:** Raffaella Colombatti, Marta Lucchetta, Maria Montanaro, Patrizia Rampazzo, Mario Ermani, Giacomo Talenti, Claudio Baracchini, Angela Favero, Giuseppe Basso, Renzo Manara, Laura Sainati

**Affiliations:** 1 Clinic of Pediatric Hematology-Oncology, Department of Child and Maternal Health, Azienda Ospedaliera-University of Padova, Padova, Italy; 2 Department of Neurosciences, Azienda Ospedaliera-University of Padova, Padova, Italy; 3 Department of Neurosciences, Neuroradiology Unit, University of Salerno, Salerno, Italy; Université Claude Bernard Lyon 1, FRANCE

## Abstract

Cerebrovascular complications are frequent events in children with sickle cell disease, yet routinely used techniques such as Transcranial Doppler (TCD), Magnetic Resonance (MRI) and Angiography (MRA), insufficiently explain the cause of poor cognitive performances. Forty children with SS-Sβ° (mean age 8 years) underwent neurocognitive evaluation and comprehensive brain imaging assessment with TCD, MRI, MRA, Resting State (RS) Functional MRI with evaluation of the Default Mode Network (DMN). Sixteen healthy age-matched controls underwent MRI, MRA and RS functional MRI.Children with SCD display increased brain connectivity in the DMN even in the absence of alterations in standard imaging techniques. Patients with low neurocognitive scores presented higher brain connectivity compared to children without cognitive impairment or controls, suggesting an initial compensatory mechanism to maintain performances. In our cohort steady state haemoglobin level was not related to increased brain connectivity, but SatO2<97% was. Our findings provide novel evidence that SCD is characterized by a selective disruption of connectivity among relevant regions of the brain, potentially leading to reduced cognition and altered functional brain dynamics. RS functional MRI could be used as a useful tool to evaluate cognition and cerebral damage in SCD in longitudinal trials.

## Introduction

Sickle cell disease (SCD) is the most common genetic disorder worldwide affecting 0.1 of 1000 newborn in non-endemic areas and around 2% of children in some African countries [1]. In the most severe forms of SCD, the homozygous SS and the double heterozygous Sβ°, the brain is frequently affected and specific alterations involve both the microcirculation and the macrocirculation. Overt ischemic stroke occurs in 11% of untreated children as a result from stenosis or occlusion in the large arteries of the Circle of Willis [2–3]. Cerebral silent infarcts (CSI), affecting 40% of children by the age of 14, are caused by small vessel disease [4–5] although recent evidence suggests that also a combination of chronic hypoperfusion or hypoxic events, favored by an underlying arteriopathy of the large vessels can lead to CSI [6]. In the past 15 years improvements have been made in the management of stroke and CSI [7]. In fact, algorithms for screening, prevention and management of stroke and CSI based on neuroimaging techniques such as Transcranial Doppler (TCD) and Magnetic Resonance Imaging/Angiography (MRI/MRA) are routinely used in clinical practice [5, 7–8]. Less progress has been made in the management of cognitive dysfunction, a further major morbidity among children and adults with SCD [3,9]. Impairment of cognitive function is reasonable in children who experienced an overt stroke or present CSIs, even at young age, due to the anatomical damage of the brain [3,10–12]. But the pathophysiology of cognitive impairment in children with SCD in the absence of structural abnormalities detected in neuroimaging studies is less clear, although limited evidence suggests that some clinical parameters such as the degree of anemia [13] and oxygen saturation [14] could be involved. In fact, patients with normal TCD and normal MRI/MRA still display cognitive deficits mainly in attention, memory and executive functions, with profound adverse impact on health, education and quality of life [3,9,10–11,15–16]. Hence, the need for biomarkers that bridge the gap between early pathophysiological alterations occurring in the brain (micro-vasoocclusion, small vessel vasculopathy, endothelial dysfunction-intimal proliferation, vascular tone dysregulation), and clinically evident impaired cognition, which seems to be a later manifestation of cerebral damage. Some cerebral abnormalities might be undetectable with conventional imaging studies [17] and the development of more sophisticated imaging techniques might reveal that MRIs which are considered normal with the currently available equipments, present subtle brain parenchymal lesions. In addition, patients with SCD could present functional brain abnormalities not evaluable by conventional studies. Functional imaging studies, whether electroencephalogram (EEG) based or MRI based, have recently been applied in SCD and have shown potential insights in exploring clinical manifestations and cognitive abnormalities in this disease [18–19].

Functional magnetic resonance imaging (fMRI) is a non-invasive imaging technique which allows the measurement of blood oxygen level dependent (BOLD) signal in different brain areas during task performance or resting state. Imaging the brain during resting state, reveals spontaneous, large-amplitude, low-frequency (<0.1 Hz) fluctuations of BOLD signal that are temporally correlated across functionally related areas [20–24]. Hence these signal fluctuations reveal the presence of functional neural networks.

With a short acquisition time, resting state fMRI can be applied in special populations such as children and patients with cognitive impairment at preclinical stages [25–28]. Some resting state networks are already present in utero, while others mature in childhood [24,27]. Around 10 major resting state networks are consistently found in adults.

One of the most widely studied functional network is the default mode network (DMN) [29]. The DMN involves the precuneus and posterior cingulate, the bilateral inferior–lateral–parietal and ventromedial frontal cortex. It seems to represent an organized, baseline mode of brain function that is suspended during any specific task-oriented behaviour and it activates when individuals are engaged in internally focused tasks such as introspection, memories retrieval and conceiving the future [23–24, 29]. DMN structures are typically deactivated during various externally focused or demanding cognitive processes. DMN alterations have been found in patients affected by Alzheimer’s disease [30], schizophrenia [31] and brain injuries [32] even before symptoms appear.

Given the abnormalities found in the DMN in other diseases with impaired cognition at a preclinical level, and our previous finding of abnormal activation in the precuneus during cognitive evoked potentials, in children with SCD independently from large vessel vasculopathy and CSI [18], we aimed at evaluating DMN connectivity by means of resting state fMRI hypothesizing that neurocognitive scores and parameters linked to cognition in SCD, such as hemoglobin [11,13] and oxygen saturation [14], could be related to brain connectivity in the DMN.

## Patients and Methods

In this cross-sectional study, consecutive children affected by SS-Sβ° SCD and regularly attending the Sickle Cell Clinic of the Azienda Ospedaliera-Università di Padova [33] were enrolled. As part of the comprehensive care program at our center patients undergo a complete check-up every year and are clinically evaluated at least every 4 months. Since 2009 the complete check-up includes: annual TCD starting at 2 years of age; MRI/MRA every two years starting when sedation is no longer necessary, generally at 5 years, and cognitive evaluation around age 5 or as soon as diagnosis is made if the child is taken in care during school years. MRIs are performed before 5 years of age in case of inability to perform TCD, indeterminate (poor window) or conditional/abnormal TCD, or clinical indication. MRI is also performed as part of pre-bone marrow transplantation evaluation. Functional MRI sequences were acquired during a standard MRI examination (see below). Since 2006 clinical and demographic information on all patients are prospectively and systematically collected in the Sickle Cell Access Database [33]. Inclusion criteria for participating in this study were: 1) brain MRI not affected by movement artifacts and 2) a detailed cognitive assessment and TCD performed within a year of MRI. Forty patients fulfilled the criteria. Previous stroke was not considered an exclusion criteria. Steady state Hemoglobin (Hb) values and day time Oxygen Saturation (SatO2) were recorded at each visit within the year of TCD, MRI and cognitive assessment. For patients on chronic transfusion the blood test was performed on the day of transfusion before the procedure.

Sixteen healthy subjects represented the control group, recruited among healthy age matched controls attending the Neurology Outpatient Clinic who performed MRI for episodic headaches.

### Ethic Statement

The medical ethic committee of our institution Azienda Ospedaliera-Università di Padova approved the study. Written informed consent was obtained from the caregivers on behalf of the children, according to the Declaration of Helsinki.

### Intellectual Function Evaluation

Intellectual function evaluation was performed on the same day of a routine hematology visit, with children in steady state. It took place in a separate and quiet room within the Pediatric Hematology-Oncology Unit. Intellectual function evaluation, conducted by licensed psychologists, was performed using the Wechsler scales [34–35]. Tests were administered according to a standardized protocol, always in the same order and were performed in Italian. Administration of the entire battery (including breaks) required 1 ½ hour- 2 hours.

The Wechsler Intelligence Scales, standard psychometric tests of general intellectual development, were chosen according to the child’s age: the Wechsler Intelligence Scale for Children-III (WISC-III) for children aged 6.7–16 years, the Wechsler Preschool Scale Intelligence (WPSSI) for those aged 4–6.6 years.

The following age-adjusted scores were reported: Full-scale IQ (FsIQ), Verbal IQ (VIQ) and Performance IQ (PIQ). The WISC-III battery of tests included also six Verbal and six Performance Subtest. The WPSSI‘s battery of tests also included five Verbal and five Performance Subtest.

Neuropsychologists and neuroradiologists were blinded to clinical findings and to each other’s results.

### Transcranial Doppler

TCD was performed using a 2 MHz pulsed Doppler ultrasonograph (EME TCD 2000/S). We used the Stroke Prevention Trial in Sickle Cell Anemia Study (STOP) criteria to assign stroke risk according to the time average mean of the maximum as normal (TAMM <170 cm/sec), conditional (TAMM 170–199 cm/sec), or high (TAMM ≥ 200 cm/sec) [36].

### MRI and MRA and fMRI

Patients and controls underwent brain MRI scans with a 1.5T MRI (Achieva; Philips Medical Systems,Best, the Netherlands) with a standard quadrature head coil. The MRI study protocol included: 3D T1-weighted imaging (TR/TE, 20/3.8 ms; flip angle, 20°; acquired voxel size, 1x1x1 mm; reconstructed voxel size, 1x0.66x0.66 mm; reconstructed matrix, 320x320; acquisition time, approximately 7 minutes); FLAIR (TR/TE/TI, 10,000/140/2800 ms; echo-train length, 53; flip-angle, 90°; slice thickness, 5 mm; acquisition pixel, 0.90x1.15 mm; reconstructed pixel, 0.9x0.9 mm; acquisition time, 3 minutes 20 seconds) and a resting state fMRI scan with 250 continuous functional volumes (TR/TE, 2216/50 ms; flip angle, 90°; 21 axial sections; acquisition matrix, 96x96; reconstructed matrix, 128x128; reconstructed pixel size, 1.8x1.8 mm; slice thickness, 5.5 mm; interslice gap, 0.5 mm; acquisition time; 8 minutes 27 seconds). During the scan, subjects were requested to remain still, stay awake, and keep their eyes open.

CSI were defined as an MRI signal abnormality of at least 3 mm in one direction and visible on two views on FLAIR T2-weighted images in a patient with normal neurological examination [37]. Volume of ischemic lesions was calculated after manually drawing the signal abnormalities on FLAIR images [∑ Area lesions × (slice thickness +interslice gap)] using a dedicated software (MedStation^®^).

Severity of occlusive changes of the internal carotid artery siphon (s-ICA), of the main segments of the anterior (A1-, A2-ACA), middle (M1-, M2-MCA) and posterior (P1-, P2-PCA) cerebral arteries was scored as follows: 0 (normal), 1 (mild stenosis), 2 (severe stenosis), 3 (occlusion) [38].

Resting-state scans were preprocessed by both Analysis of Functional NeuroImages (version AFNI_2010_10_19_1028; http://afni.nimh.nih.gov/afni; NIMH, Bethesda, Maryland) and FM-RIB Software Library (version FSL 4.1.9; http://www.fmrib.ox.ac.uk; FMRIB). Preprocessing was performed as described in Biswal et al and in Neuroimaging Informatics Tools and Resources Clearinghouse (www.nitrc.org/projects/fcon_1000).

Nine SCD patients and 4 controls displayed a single brief movement of head displacement > 3mm or 3° during scanning. We decided to remove the interested volumes (up to 10 volumes) before undergoing preprocessing. Control temporal-concatenation group ICA analysis was used to generate 25 group-level components of the dataset by Multivariate Exploratory Linear Optimized Decomposition into Independent Components (MELODIC, FSL) [39]. We decided to consider only the control subjects to better identify the networks, since we speculated that the presence of possible parenchymal alterations secondary to SCD might hinder the correct network recognition. Before statistical inference, we identified correctly the DMN, by visual inspection and by comparison with available maps in the literature [40].

The dual-regression approach was used to obtain a connectivity map of the DMN in each subject. The standardized map obtained by dual regression was used to perform group comparisons. Nonparametric permutation testing (5000 permutations) was used for statistical analysis of spatial maps, by the TFCE method for multiple comparisons and thresholding at p<0.05.

### fMRI Group Comparison

fMRI group comparison was performed at first comparing SS-Sβ° patients vs controls, then in 6 subgroup analysis considering: the results of neurocognitive evaluation, the degree of anemia, the daytime SatO2, the presence of CSI on MRI, the presence of stenosis at MRA, the conditional/abnormal velocities at TCD.

Patients were subdivided according to the results of neurocognitive evaluation, for FsIQ, VIQ and PIQ level: ≥ 75 was considered normal. Group comparisons were then performed between patients with normal vs abnormal values and then each group vs controls.

In order to be sure that anemia did not influence BOLD signal, we performed comparison according the degree of anemia: patients with mean steady state Hb level ≥ 8mg/dL vs controls, patients with mean steady state Hb level < 8mg/dL vs controls, patients with steady state Hb level ≥ 8mg/dL vs those with Hb level < 8mg/dL.

A comparison was performed in patients according to daytime SatO2 ≥97% versus <97%.

We subdivided SS-Sβ° patients according to TCD examination results, comparing groups with normal, abnormal/conditional and low picture vs controls and among each other.

For MRI we considered patients with CSI and without CSI. We then evaluated role of lesion volume on connectivity. For MRA we considered patients with stenosis compared to patients without stenosis.

## Results

Forty patients (39 SS, 1 Sβ°), 21 M 19 F, mean age at brain MRI 8.08 ± 2.83 years (range 4.6–15) were enrolled. Thirty eight patients (95%) were Africans (24 Nigeria, 5 Ghana, 3 Congo, 3 Senegal, 1 Cameroon, 1 Burkina Faso, 1 Togo) and 2 from Central America. Main clinical and demographics characteristics are shown in Table 1. None of the patients had suffered a stroke and neurological examination was normal in all. Mean steady state Hb levels were ≥ 8 mg/dL in 30/40 (range 7.1–10.6 mg/dL) and steady state daytime SatO2 was≥97% in 31/40 (range 95–100%). At time of MRI, 27 patients (67,5%) were not receiving any disease modifying treatment; nine were on chronic transfusion (6 exchange transfusion, 3 top-up transfusion) for abnormal TCD (n.7) and for lack of response to hydroxyurea (n.2) while 4 were receiving hydroxyurea for recurrent acute chest syndromes (n.3) or previous chronic anemia <7 g/dl (n.1).

**Table 1:** Patients’ demographics, clinical characteristics and neurocognitive scores. *Only in patients performing WISCIII.

	N° of Patients or Mean (range)
**Phenotype**	
SS	39
Sβ°	1
**Gender**	
M	21
F	19
**Age** (years)	8,08 (4,6–15,0)
**Neurocognitive evaluation**	
Full scale IQ	88,2 (55–125)
Verbal IQ	83,2 (52–123)
Performance IQ	96 (60–121)
**Verbal Subtests**	
Information/General Culture	8,00 (3–12)
Similarities	8,84 (2–14)
Arithmetic	7,22 (1–12)
Vocabulary	7,50 (3–13)
Comprehension/ General Comprehension	5,79 (1–12)
Digit Span/Phrases*	7,91 (2–15)
**Performance Subtests**	
Picture Completion	11,14 (3–15)
Coding/Animal House	9,08 (6–18)
Picture Arrangement/Retest Animal House*	8,62 (4–11)
Block Design	9,15 (2–15)
Object Assembly/ Geometric Design	9,41 (4–14)
Symbol Search*	9,08 (1–15)
Mazes	9,56 (1–17)
**Hemoglobin** (g/dL)	8,5 (7,1–10,6)
**O2 pulse oxymetry**	97,78 (95–100)

Sixteen healthy controls, 5 M 11 F, had mean age of 9.98±2.8 (range 5–15).

### Standard Neuroimaging Techniques: Transcranial Doppler, MRI/MRA

All patients had normal TCD at time of MRI scan. Nevertheless, 7/40 (17,5%, mean age 7.83± 3.26 years, 7 females) had a history of abnormal TCD.

MRI was negative in 20/40 (50%). The remaining patients presented CSI in the white matter with a mean lesion volume of 1471,05 mm3 (range 30–5412). MRA was negative in 12/40 (30%), with single or multiple stenosis in the remaining patients; none had vessel occlusion.

MRI and MRA were normal in all controls.

### Cognitive Evaluation

FsIQ was normal in 33/40 patients (82.5%, mean age 8.32 ± 2.83 years; 16 females) and <75 in 7/40 (17.5%, mean age 9 ± 4. years; 3 females), revealing cognitive impairment.

VIQ was normal in 29/40 patients (72.5%, mean age 8.36 ± 3.17 years; 14 females) and <75 suggesting compromise of the vebal domain in 11 (25%, mean age 8.62 ± 2.92 years; 5 females).

PIQ was normal in 35 children (87.5%, mean age 8. 25± 2.82 years; 17 females), and <75 in 5 (12.5%, mean age 9.7 ± 4.71 years; 2 females).

Mean scores of FsIQ, VIQ, PIQ and all subtests are shown in Table 1.

### Default Mode Network Analysis with Resting State Functional MRI

**Fig 1:**
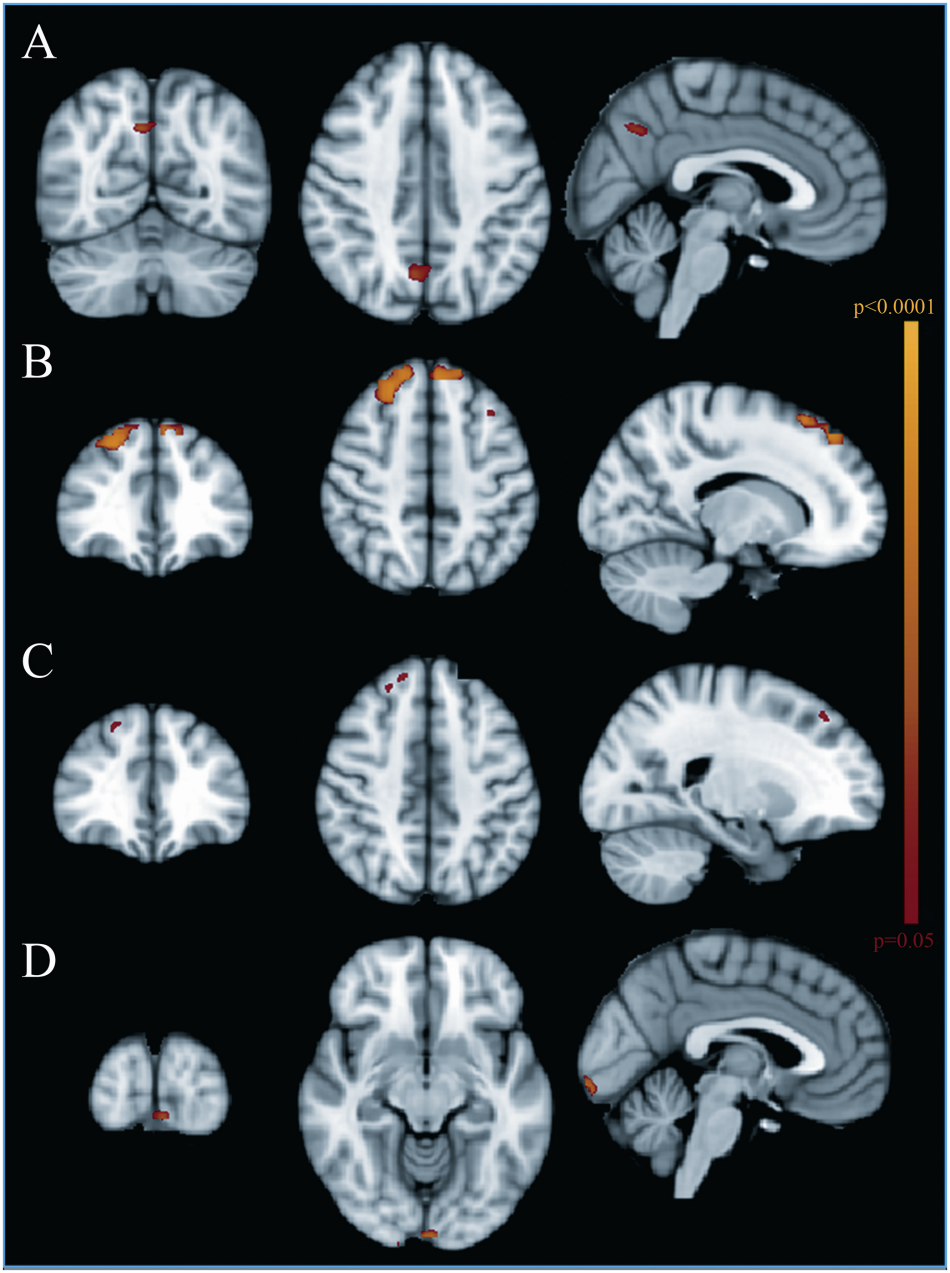
Functional MRI group analysis. (A) Increased connectivity in the Default Mode Network (DMN), in the posterior precuneus, in patients versus controls; (B) Increased connectivity in the DMN, in the medial prefrontal regions, in patients with normal Verbal IQ compared to controls; (C) Increased connectivity in the DMN, in the right prefrontal region, in patients with normal MRI compared to controls; (D) Increased connectivity in the posterior calcarine region in patients with Oxygen Saturation≤97% compared to patients with higher Oxygen Saturation.

**Fig 2:**
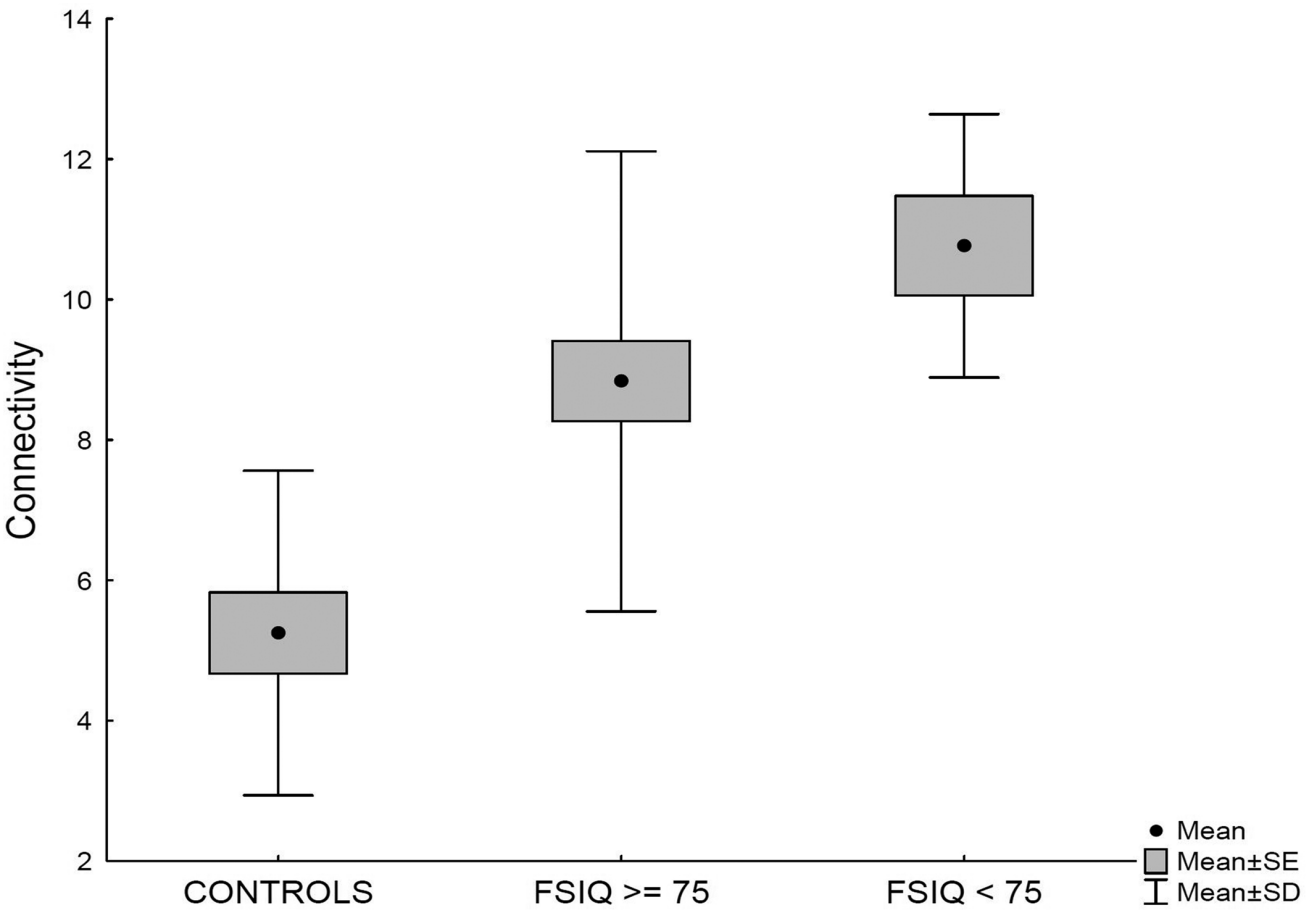
Connectivity values in the Precuneus in controls, patients with preserved cognition, i.e. normal FsIQ (FsIQ≥ 75), patients with impaired cognition, i.e low FsIQ (FsIQ<75).

Children with SCD presented significant increased connectivity in a small area of the DMN located in the posterior precuneus (cluster size 34 voxels, peak 28, 21, 37, Fig 1A) compared to controls. Although subgroup comparison (controls, SCD children with FsIQ≥75, SCD children with FsIQ<75) did not reveal significant differences in this area, the mean connectivity within the cluster showed an increasing connectivity from controls, to SCD children with normal cognition and SCD children with low FsIQ (Fig 2).

VIQ and PIQ comparison showed no differences among SCD patients, although SCD patients with normal VIQ presented a higher connectivity compared to controls in medial prefrontal regions (3 clusters, n.1: size 211 voxels, peak 22, 53, 40; n.2: size 200 voxels, peak 32, 56, 40; n. 3 size 6 voxels, peak 18, 46, 42, Fig 1B).

Among SCD patients, there was no connectivity difference according to CSI lesion volume, although SCD patients with normal MRI showed a small cluster of increased connectivity in the right prefrontal region (cluster size 23 voxels, peak 25, 57, 39, Fig 1C) compared to controls.

No significant difference in connectivity could be found between SCD patients with normal TCD and those with a history of abnormal TCD nor according to the presence and degree of stenosis at MRA.

Among SCD patients, there was no DMN connectivity difference according to steady state hemoglobin levels, while a SatO2≤97% was associated with increased connectivity in a small area located in the posterior calcarine region (Fig 1D).

## Discussion

To our knowledge, our study is the first to investigate the DMN in children with SCD by means of resting state fMRI revealing significant functional changes early in the disease course. In particular, our study revealed increased connectivity within the precuneus in patients compared to controls, more evident among those with decreased cognitive performances. Since the DMN is involved in speculative activities such as memory and planning, its connectivity results decreased in patients with severe cognitive impairment [29–31], but can be increased at a preclincal stage (low or mild impairment). In fact, there is a growing evidence of a compensatory role of the connectivity changes in the early stages of cognitive failure [41–42]. Therefore, the increased connectivity in the precuneus of children with SCD likely represents a similar phenomenon, i.e. the recruitment of the DMN for counterbalancing the progressively emerging cognitive deficits. Interestingly, the precuneus has been shown to present also morphological changes in children with SCD: the precuneus and the posterior cingulate gyrus showed progressive thinning [43–44], and this finding was interpreted as a consequence of regional vulnerability to decreased oxygen delivery in relation to an impaired blood flow. According to our functional data, cortical thickness changes in SCD might result from an increased functional demand combined to an impaired blood flow indicating that the connectivity modulation in areas of the DMN might help to preserve cognitive functions but also induce a progressive regional cortical exhaustion. The absence of significant differences between SCD patients subgroups (normal FsIQ and low FsIQ) vs controls likely reflects the decreased statistical power of small samples and prompts for larger population studies on SCD children and for longitudinal studies evaluating cognition and DMN connectivity changes.

Regarding the functional involvement of the DMN in SCD, cortical sources of abnormal EEG activity in the precuneus [18] in SCD have already been demonstrated, indicating that the brain areas of the DMN (precuneus, posterior cingulate, the bilateral inferior–lateral–parietal and ventromedial frontal cortex) are critically involved in this condition. A similar mechanism of compensation through increased connectivity might occur to maintain the VIQ thus explaining the changes detected in the medial prefrontal regions of the DMN in patients with low VIQ. Nevertheless, language abilities could be better evaluated with specific networks of the dominant hemisphere that require a dedicated analysis of areas specifically involved in language functions.

The reasons of connectivity changes within the DMN remain elusive and confounding factors such as chronic anemia or decreased SatO2 need to be investigated. Interestingly, there was no significant alteration in DMN connectivity in patients with more severe anemia, showing that BOLD signal is not influenced by a low steady state Hb level ≥7 g/dL. Chronic anemia (Hb< 7 g/dL or Ht<20%) is a strong independent risk factor for both CSI [6] and cognitive impairment in the presence of normal MRI [3,13,45]. None of the children in our cohort had a mean steady state Hb<7 g/dL which might justify why there was no difference in brain connectivity according to the degree of anemia in our cohort. Nevertheless, our previous analysis using functional EEG based techniques and analysis of cortical sources of cognitive evoked potentials also failed to demonstrate a correlation with the degree of anemia or a history of anemia in the first 5 years of life, suggesting that anemia alone might not be sufficient to determine impaired cognition in SCD [18]. Some of the patients were on chronic transfusion and this also could have contributed in reducing the importance of Hb value in our analysis. Further studies in larger cohorts would be useful to compare the effect of disease modifying treatments such as HU or chronic transfusions on brain connectivity changes.

Low daytime SatO2, on the contrary, was significantly related to the connectivity of the DMN in our cohort, but revealed connectivity changes in a very limited areas outside the regions that were significantly involved by cognitive performances. Children with lower daytime mean steady state SatO2 presented increased connectivity, with possibly the same mechanism of an initial attempt to compensate the lower delivery of O2 to the tissues. In fact, SatO2 is a clinical factor related to cognition [14,46] and was recently related to white matter damage other than CSI [47]. Probably, the lower O2 delivery to the cerebral tissue, as documented by low SatO2, is only one of the means through which cortical disruption can occur.

The increased connectivity seems to be unrelated to large vessel vasculopathy alone because there was no difference in connectivity according to history of abnormal TCD or presence of stenosis on MRA. Moreover the increased connectivity was also unrelated to CSI. These data might indicate a preclinical alteration in brain connectivity, undetectable by standard neuroimaging. This data suggests that abnormal brain function might begin particularly early in children with SCD, considering the relatively young age of our population (8 years). It might implicate also that cognition in SCD is influenced more by factors limiting O2 delivery to the tissues than by CSI or large vessel vasculopathies. In this sense, correlation of nighttime SatO2 with connectivity and interventions aimed at increasing SatO2 could be significant [48].

Finally, the percentage of CSI and stenosis in our population was higher compared to other cohorts both in the United States and Europe. The majority of our patients were from West Africa and it could be that ethnic differences could play a role in the variability of cerebral vasculopathy in children with SCD as it happens in stroke for adults [49].

## Conclusions

In conclusion, resting state fMRI analysis reveals functional abnormalities in brain connectivity that seem independent from the known mechanisms of cerebral vasculopathy in SCD, such as large vessel disease or CSI and are thus not revealed by routinely used imaging techniques. fMRI, together with other functional EEG imaging studies, might represent a useful complimentary analysis for investigating the disease natural course in studies linking physiopathology, anatomical and functional changes in the DMN. In addition, as neural network connectivity might be positively influenced by behavioral, educational or environmental factors [50], children with SCD could benefit from specific treatments and their response could be closely monitored by resting state fMRI up to cognition improvement.
